# Custom stems for femoral deformity in patients less than 40 years of age: 70 hips followed for an average of 14 years

**DOI:** 10.3109/17453670903062470

**Published:** 2009-08-01

**Authors:** Michael Akbar, Guenther Aldinger, Knut Krahmer, Thomas Bruckner, Peter R Aldinger

**Affiliations:** ^1^Department of Orthopedic Surgery, University of HeidelbergGermany; ^2^The Paulinenhilfe Orthopedic Center, DiakonieklinikumStuttgartGermany; ^3^Department of Medical Biometry and Informatics, University of HeidelbergHeidelbergGermany

## Abstract

**Background and purpose** Femoral deformity associated with osteoarthritis is a challenge for both the surgeon and the implant. Many of the patients with these deformities are young. Standard implants can be difficult to fit into these femurs. We prospectively evaluated the outcome of custom uncemented femoral stems in young patients.

**Methods** 61 consecutive patients (72 hips) underwent surgery for osteoarthritis because of femoral deformity at a mean age of 35 (22–40) years. The patients received a CT3D-A custom-made femoral stem and an uncemented cup. The mean follow-up time was 14 (10–16) years. 2 patients died at 7 and 8 years after surgery, otherwise, none of the patients were lost to follow-up.

**Results** At follow-up, the femoral prosthesis had not been revised in 59 patients (70 hips). 3 patients (3 hips) had required revision surgery due to loosening of the acetabular component; 2 hips were awaiting revision surgery for loosening of the acetabular cup. There were no cases of dislocation or infection. At review, all stems were considered stable according to the radiographic criteria. No migration or subsidence was observed on plain radiographs.

**Interpretation** Our results are comparable to published results of custom stems regarding survival and outcome. Considering the young age and the deformities in this series of uncemented custom femoral stems, and the fact that there was follow-up of up to 16 years, the survival is remarkable. This technique appears to be a reasonable alternative in younger patients with femoral deformities.

## Introduction

In femoral deformities, primary stability can be difficult to achieve with standard stems (Berry 1999). Because of the large anatomical variation in the proximal femur at different locations (Reuben et al. 1992, Bert 1996), optimal fit-and-fill of the metaphysis is difficult to achieve with commercially available prostheses, even considering the variety of anatomical designs and sizes (Wettstein et al. 2005). Anatomical variation of the proximal femur affects not only the intramedullary cavity but also the extramedullary parameters, such as the neck-shaft angle (center-collum-diaphyseal (CCD)), offset, anteversion, and the helitorsion angle (Husmann et al. 1997, Berry 1999). The development of custom uncemented hip stems designed with regard to intramedullary and extramedullary parameters has proven useful in the treatment of dysplastic hips (Gau et al. 1988), in osteoarthritis secondary to congenital dislocation (Flecher et al. 2006), and in primary osteoarthritis (Wettstein et al. 2005). For patients with femoral deformity and a long life expectancy, an approach using custom-designed, uncemented hip prostheses may represent a useful alternative to the current generation of cemented stems or anatomic, uncemented prostheses.

Here we report on a series of 3-dimensionally custom, uncemented femoral stems inserted in young patients with femoral deformities, with a minimum follow-up of 10 years.

## Patients and methods

In our tertiary arthroplasty referral center, 2,367 hip arthroplasties were performed between 1992 and 1994. In 1,123 hips, a custom femoral component was used: in 329 of these hips this was for severe femoral deformity. 61 patients (72 hips) were under the age of 40 years at the time of surgery and all were included in the present prospective study. The study was approved by the Institutional Review Board of the University of Heidelberg (346/ 2004 and 081/2005) and the study was carried out in accordance with the Helsinki Declaration of 1975.

The series comprised 72 uncemented total hip arthroplasties in 61 patients (33 men), all of whom were younger than 40 years (mean age 35 (22–40) years) with femoral deformities and osteoarthritis (Table). 5 men and 6 women had bilateral surgery at different sessions. Mean body weight was 77 (50–140) kg, the mean height was 171 (150–196) cm, and mean BMI was 26 (18–41). All patients were evaluated prospectively and operated on between 1992 and 1994 by 4 surgeons at our center.

**Table:** Patient demographics, diagnoses, and classification of proximal femoral deformity

	Hips (patients)
Sex	
Male	38 (35)
Female	34 (28)
Body mass index (BMI), mean [SD]	26 [5]
Diagnosis	
Dysplasia (DDH)	25
Hip dislocation (CDH)	8
Avascular necrosis (AVN)	8
Osteoarthritis (OA)	2
Posttraumatic OA	12
Perthes’ disease	8
Rheumatoid arthritis (RA)	7
Slipped epiphysis	2
Previous osteotomies	34
Classification of proximal femoral deformity	
Site of deformity	
Greater trochanter	27
Femoral neck	35
Metaphysis	29
Diaphysis	15
Geometry of deformity	
Torsion	24
Angular	35
Translational	28
Size abnormality	12

**Figure 1.**
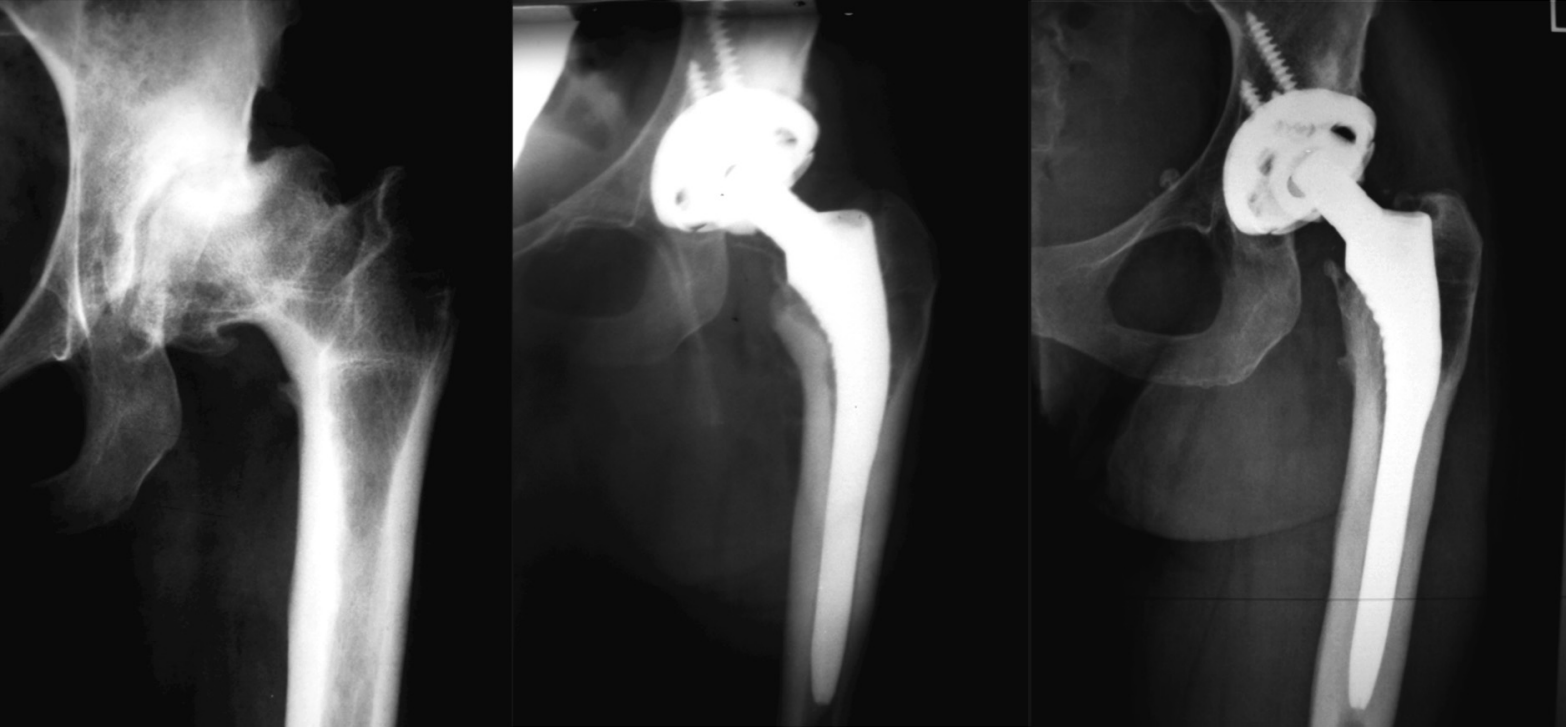
26-year-old woman with severe secondary OA of the left hip, femoral deformity, and varisation osteotomy. Radiographs (left to right) preoperatively, and 1 year and 15 years postoperatively.

All patients had a femoral deformity according to the criteria of Berry (1999). The distortion of the femoral morphology was the indication for using the custom stem in this study. All patients received a CT3D-A custom-made femoral stem (OS Orthopedic Services GmbH, Mainhausen, Germany) (Figure 1) and a Double-Spherical-Pressfit cup (DSP-CUP) (OS Orthopedic Services) with a polyethylene (PE) insert and a 28-mm ceramic head (Ceramtech, Plochingen, Germany). PE inserts that had been gamma-irradiated in gas with a minimum of 6 mm thickness were used in all cases.

Design of the custom CT3D-A stem uses computer technology based on CT images. For construction of the implant, the femur was first reconstructed in three dimensions. The hip stem is derived directly from the form of the bone cavity. The outer and inner surfaces of the hard bone of every patient are reconstructed three-dimensionally. The important area for the prosthesis design is the strong trabecular transition structure between corticalis and cancellous bone. Threshold contouring was used with 500 Houncefield units as a threshold value.

The finding of the contours is done automatically; however, any irregularities such as disturbing bone lamellae or possible interruptions in the bone can be corrected interactively by the design engineer.

This provisional implant was then modified progressively using a computer program supplied by the manufacturer, with which the stem is introduced in a virtual model and extracted from the femur while maintaining preselected areas of bone contact for the best filling and fitting in the proximal metaphysis. Distal diaphyseal fixation was avoided by reducing the diameter of the stem. The length of the stem ranged from 140 to 160 mm. The extramedullary part of the prosthesis is designed for restoration of a physiological CCD angle, neck offset, and anteversion. The final shape of the prosthesis was transferred to a computer-assisted machining (CAM) device and the stem was prepared from a titanium block (TiAl6V4). The macro-structure with a medial bridge and arched structure effectively strengthen both the axial and the rotational stability. A coating layer of hydroxyapatite (HA) (thickness, 80–150 µm) was applied to the proximal two-thirds of the implant. The program also constructs the corresponding rasp for each stem individually.

The 3-D preoperative planning was carried out during the design process by the engineer and was validated by the surgeon. This planning laid special emphasis on the neck osteotomy level, the final position of the implant in relation to the lesser and greater trochanters, and the values of helitorsion and neck anteversion.

An anterolateral approach was used for all hips. The femoral cavity was prepared using the custom broach that mimicked the shape of the stem. A pneumatic hammer was used in all cases for preparation of the femur to achieve compaction of the cancellous bone, so that the prosthesis would fit at the correct level. All 72 stems were implanted according to the preoperative plan without any problems or complications intraoperatively. No fracture of the femur, incorrect fit of the prosthesis, or incorrect torsion of the neck of the prosthesis was observed during the surgical procedures. All patients started walking on the day after surgery with full weight bearing as tolerated.

During follow-up, 2 patients died of unrelated causes (7 and 8 years after surgery). Thus, 59 patients (70 hips) were reviewed clinically and radiographically by two independent observers at a mean follow-up time of 14 (10–16) years. For 55 of these patients, the length of follow-up was 12 years or more. No patients were lost to follow-up. All results were analyzed clinically on the basis of preoperative and postoperative Harris hip score (HHS). We assessed patients subjectively by asking them how they felt about their procedure (dissatisfied, satisfied, or very satisfied). The pre- and postoperative activity levels of the patients were assessed according to Devane et al. (1997). Clinical assessment included limp, range of motion, and pain. Patients assessed their pain in the hip after surgery at the time of follow-up on a visual analog scale (VAS; 0–10). The range of motion of the hip joint was measured with a goniometer. The “full range of motion” of the hip joint was defined as flexion > 90°, abduction > 15°, adduction > 15°, internal rotation > 15°, and external rotation > 15°.

At the time of review, AP and lateral radiographs of the hip were compared with those taken immediately after surgery and with those taken regularly during the postoperative follow-up. Two independent, experienced orthopedic surgeons compared the radiographs for stem alignment, subsidence, radiolucent lines, bone hypertrophy, osteolysis, stress shielding, pedestal formation of the stem tip, heterotopic ossifications, and femoral and acetabular loosening. Radiolucent lines, endosteal ossifications, and osteolysis were recorded for the seven Gruen zones and bone hypertrophy was defined as thickening of the periprothetic diaphyseal bone. Osteolysis was defined as areas of localized bone resorption or endosteal erosion. Stress shielding was defined according to Engh et al. (1987). Pedestal formation was defined as a shelf of endosteal new bone at the stem tip partially or completely bridging the intramedullary canal. Radiographic failure was defined as subsidence of the stem of more than 2 mm, variation in the frontal stem axis of more than 2°, any osteolysis, or any radiolucency of more than 2 mm—or that progressed with time. Acetabular loosening was defined as continuous migration of > 5 mm or tilting of > 5° compared with baseline AP radiographs. Criteria for stability were the same as those described by Engh et al. (1990).

### Statistics

Kaplan-Meier survival analysis was performed with two endpoints: (1) revision procedures of the acetabular component and of the stem for aseptic loosening, and (2) revision procedures of the acetabular component and the stem for any cause. The statistical analysis was done with SAS software version 9.1 for Windows (SAS Institute Inc., Cary, NC).

## Results

### Revisions, survival analysis, and complications

**Figure 2.**
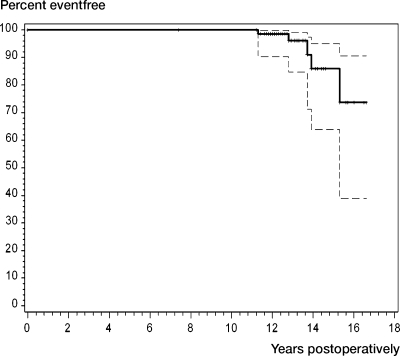
Kaplan-Meier survival curve for all revisions (only cup revisions occurred).

Cup failure occurred in 5 of the 70 hips: 3 hips had required revision for cup loosening and 2 patients were awaiting revision surgery for cup loosening. The cups showed an overall survival rate of 100% after a mean follow-up time of 10 years (number at risk 70), of 99% (CI: 90–100) after a mean follow-up time of 12 years (number at risk 55), and of 86% (CI: 64–95) after a mean follow-up time of 14 years; however, the number at risk had dropped to 15 at this point (Figure 2).

None of the femoral prostheses were revised. There were no cases of dislocation. 2 temporary nerve palsies and 1 case of a fissure in the greater trochanter healed without treatment.

### Clinical results

The mean preoperative HHS of the 70 hips that were available for follow-up was 41 (17–58). The mean HHS of these hips at the time of follow-up evaluation was 87 (42–100) points (p < 0.001). None of the patients reported start-up pain or thigh pain during walking. The mean preoperative gait score was 11 (7–15) (of a possible 33 points) and increased to 29 (25–30) at follow-up (p < 0.001). At the latest follow-up, 54 patients could walk 1 km or more. 1 patient used a cane for longer distances and 3 patients always used a cane.

Preoperatively, most of the patients had a low level of activity according to Devane et al. (1997) (mean 2.6 (1–3)). At the latest follow-up, the mean activity level was 3.8 (2–5) (p < 0.001) and 65 of the 70 hips had a full range of motion. No pain was reported for 44 hips; 19 hips gave slight pain, 6 hips gave moderate pain, and 1 hip gave severe pain (caused by a loose cup; listed for revision). Subjectively, 68 patients were satisfied. 2 patients were dissatisfied due to cup loosening.

### Radiographic evaluation

There was no radiographic evidence of loosening or subsidence in any of the femoral stems. The positioning of the stems at the time of follow-up was neutral in 69 hips; 1 hip had a varus position. 3 hips (3 patients) had localized radiolucent lines in Gruen zones 1 and 7 without progression over time; femoral osteolysis was not found in any case. Severe stress shielding (2° to 4°) was not observed. Distal femoral hypertrophy was limited to 1 zone in all cases and observed in Gruen zone 2 (6%), 3 (16%), 5 (37%), or 6 (7%) in 46 of the 70 hips. Pedestal formation at the tip of the prosthesis was observed in x hips. All stems were considered stable according to the criteria of Engh et al. (1990). There was evidence of radiographic loosening in 5 cups in 5 patients. 3 of these cups had already been revised. 22 patients had asymptomatic heterotopic ossifications, 19 patients (21 hips) with grade I, 2 with grade II, and 1 with grade III according to Brooker et al. (1973).

## Discussion

Most femoral deformities are found in young patients and are due to posttraumatic OA, dysplasia, congenital hip dislocation, or to congenital deformities (Berry 1999). Because of the large anatomical variation in the femoral geometry at different locations, close-fitting femoral components cannot be consistently achieved with standard commercially available implants (Wettstein et al. 2005). In order to solve this geometrical mismatch between the femoral canal and uncemented implants, custom-made femoral stems were initially developed in the 1980s (Aldinger et al. 1983) and refined in the 1990s as computer technology became more widely used (Aamodt et al. 2001). Here we report on a series of 3-dimensionally computed custom stems in a subgroup of high-risk patients (i.e. young patients with deformity).

The technique involved custom femoral stems, whereby the prosthesis was optimally fitted to the femoral cavity and the extramedullary joint anatomy of each patient was reconstructed according to CT data in order to help to reduce prosthetic impingement and dislocation, and to improve joint mechanics.

No femoral stem revisions were required up to 16 years postoperatively; similar good results have been reported in other series of uncemented and cemented stems in young patients (Ballard et al. 1994, Dorr et al. 1994, Berry 1999, Aldinger et al. 2003). The scarcity of arthroplasty results in very young patients makes comparison of different studies difficult, as the diagnoses and deformities vary between studies. We have found only one report on custom femoral stems for severely distorted proximal anatomy of the femur in young patients; this involved 48 hips with a 6- (4–8) year follow up. All implants had optimal fit and fill. None of the patients had thigh pain and none had aseptic loosening of the implant components (Koulouvaris et al. 2008).

The custom implants in our series were HA-coated. Satisfactory survival rates with HA-coated femoral stems have been reported in adult patients; Capello et al. (2003) reported a stem failure rate of 2% in patients below the age of 45 years when using a proximally HA-coated implant, with a minimum follow-up of 10 years.

Despite the moderate cup loosening rate of 5 cups in 72 hips, we did not find any progressive femoral osteolysis after a mean follow-up of 14 years. This is a remarkable finding, as the prevalence of osteolysis in uncemented prostheses has been reported to vary between 40% and 60% in younger age groups (Kawamura et al. 2001). The low degree of proximal bone loss in our study might indicate a rather physiological weight distribution from the stem to the femoral bone, but we cannot prove this. Published evidence suggests that third-generation cemented fixation of the cup still has superior survival in large subgroups of populations studied; however, survival of uncemented implants continues to improve with new bearing options (Morshed et al. 2007).

Cortical hypertrophy was localized in Gruen zone 2 (6%), 3 (16%), 5 (37%), or 6 (7%). The design of the prostheses with a proximal fit-and-fill could explain this pattern of cortical hypertrophy, because the stress is mostly transmitted from the prosthesis to the cortical bone in the proximal metaphysis (Wettstein et al. 2005). The stem is, however, shorter than the majority of standard stems; this may have led to a more proximal load transfer.

The long-term clinical results of our series of young patients concerning function, activity level, postoperative need for walking support, and patient satisfaction are similar to the results for patients with standard and custom prostheses (Kusswetter and Sell 1993, Bert 1996, Smith et al. 2000, Malchau et al. 2002, Aldinger et al. 2003, Wettstein et al. 2005, Grubl et al. 2006).

We have found very few reports on hip prostheses with commercially available femoral stems in young patients, and with medium-term or long-term follow-up (Malchau et al. 2002, Aldinger et al. 2003, Grubl et al. 2006), and even fewer series involving custom femoral stems with medium-term follow-up (Kusswetter and Sell 1993, Bert 1996). In series with different types of custom femoral stems, thigh pain rates of between 0% and 17% and loosening rates of between 0% and 35% have been reported (Kusswetter and Sell 1993, Bert 1996, Wettstein et al. 2005) .

Results with customized cementless stems have not been any better, and sometimes worse, than results with commercially available stems (Wettstein et al. 2005). This might be explained by the fact that their design has often been based on radiographs, which cannot be used for precise assessment of the morphological 3-D features of the femur. Bargar (1989) reported that design based on 2-D CT scans and radiographs is insufficient for anatomical fit of the prosthesis to the bone, because it is based on a 2-D concept. The implant can be better adapted to the morphological features of bone by using CT of the femoral anatomy, resulting in improved results in various follow-up studies (Kusswetter and Sell 1993, Wettstein et al. 2005) and in our series. However, the use of a CT scan alone is insufficient for precision-design of a customized femoral stem.

Our series and some others (Kusswetter and Sell 1993, Wettstein et al. 2005) are notable because 3-D reconstruction of the femur was used to shape the prosthesis and the joint geometry. Based on the results, this procedure appears to be more accurate than conventional of the shelf stems.

The cost of a custom-made femoral stem is about twice that of a standard, uncemented stem (Wettstein et al. 2005). However, the technique appears to be a reasonable alternative in younger patients with femoral deformities.
